# Genome-wide insights into population structure and host specificity of *Campylobacter jejuni*

**DOI:** 10.1038/s41598-021-89683-6

**Published:** 2021-05-14

**Authors:** Lennard Epping, Birgit Walther, Rosario M. Piro, Marie-Theres Knüver, Charlotte Huber, Andrea Thürmer, Antje Flieger, Angelika Fruth, Nicol Janecko, Lothar H. Wieler, Kerstin Stingl, Torsten Semmler

**Affiliations:** 1 Genome Sequencing and Genomic Epidemiology, Robert Koch Institute, Nordufer 20, 13353 Berlin, Germany; 2 Advanced Light and Electron Microscopy, Robert Koch Institute, Berlin, Germany; 3 Department of Electronics, Information and Bioengineering, Politecnico di Milano, Milan, Italy; 4 Department of Mathematics and Computer Science, Freie Universität, Berlin, Germany; 5 National Reference Laboratory for Campylobacter, Department of Biological Safety, German Federal Institute for Risk Assessment, Berlin, Germany; 6 Methodology and Research Infrastructure, Robert Koch Institute, Berlin, Germany; 7 Division of Enteropathogenic Bacteria and Legionella, Department of Infectious Diseases, National Reference Centre for Salmonella and Other Bacterial Enterics, Robert Koch Institute, Wernigerode, Germany; 8 Microbes in the Food Chain, Quadram Institute Bioscience, Norwich, UK

**Keywords:** Computational biology and bioinformatics, Ecology, Microbiology

## Abstract

The zoonotic pathogen *Campylobacter jejuni* is among the leading causes of foodborne diseases worldwide. While *C. jejuni* colonises many wild animals and livestock, persistence mechanisms enabling the bacterium to adapt to host species' guts are not fully understood. In order to identify putative determinants influencing host preferences of distinct lineages, bootstrapping based on stratified random sampling combined with a *k-mer-*based genome-wide association was conducted on 490 genomes from diverse origins in Germany and Canada. We show a strong association of both the core and the accessory genome characteristics with distinct host animal species, indicating multiple adaptive trajectories defining the evolution of *C. jejuni* lifestyle preferences in different ecosystems. Here, we demonstrate that adaptation towards a specific host niche ecology is most likely a long evolutionary and multifactorial process, expressed by gene absence or presence and allele variations of core genes. Several host-specific allelic variants from different phylogenetic backgrounds, including *dna*E, *rpo*B, *ftsX or pyc*B play important roles for genome maintenance and metabolic pathways. Thus, variants of genes important for *C. jejuni* to cope with specific ecological niches or hosts may be useful markers for both surveillance and future pathogen intervention strategies.

## Introduction

*Campylobacter jejuni* is regarded as a common resident among the gut microbiota of many wild and agriculture-associated animals^1^, especially birds, poultry and cattle^2,3^. Contamination of (chicken) meat, water, raw-milk and other food products along the food production chain is therefore the most attributable factor of diarrheal disease caused by *C. jejuni* in humans^3–6^. As a result *C. jejuni* is a bacterium frequently isolated from human patients suffering from acute gastroenteritis^6^.

Previous research using multilocus sequence typing (MLST) of *C. jejuni* from different origins showed that specific sequence types (STs) were frequently associated with a particular host species^7^. While STs belonging to the clonal complexes (CC)-42 and CC-61 are common among *C. jejuni* of cattle and/or other ruminate origins, STs belonging to CC-257, CC-353 or CC-1034 are regarded as chicken-specific^8–10^. Isolates belonging to STs sharing a clonal complex such as CC-21, CC-45 or CC-48 commonly occur in samples of multiple host species, indicating the ability of these phylogenetic lineages to rapidly switch between different (intestinal) conditions, and, therefore, representing a typical host-generalist lifestyle^11^. Factors influencing adaptation of *C. jejuni* to certain host species, especially to poultry and cattle, were an important focus of *Campylobacter* research over the last decade^12–14^. In recent years, novel bioinformatic methods and tools such as genome-wide association studies (GWAS) proved their potential to identify genetic factors promoting host adaptation and/or pathogenicity in *C. jejuni*^13–17^. For instance, accessory genes encoding factors involved in the bacterial vitamin B5 biosynthesis pathway were found to be associated with cattle and its typical diet^13^, while proteins enhancing iron acquisition abilities of the bacteria during infection were harboured by isolates from human clinical samples ^16^. Previous studies employing GWAS often implemented a gene-by-gene approach for population scale analysis or focused on particular strains, such as CC-45^13,15,16^, a phylogenetic background known for its frequent association with cases of human diseases worldwide^14,18–20^ .

Most of these GWAS have been predominately focused on the variable set of genes commonly addressed as accessory genome. However, changes among (essential) core genes (i.e. basic cellular and regulatory functions) within the *C. jejuni* population may reflect adaptation towards a particular bacterial lifestyle as well.

Core genome alterations are thought to play an important role in overcoming specific host-associated intestinal stress conditions^21,22^, while other alterations may enable certain *Campylobacter* lineages to cope with colonisation inhibitors or even diets associated with gastrointestinal tracts of a much broader range of host species^23^. A recent GWAS study indicated that the worldwide intensified cattle farming for meat production was accompanied by a timeline of genomic events enhancing host adaptation of certain *C. jejuni* lineages to cattle^24^.

The aim of this study was to generate in-depth insights into the current population structure of *C. jejuni* by using high resolution of whole genome sequencing and a stratified random sampling approach combined with GWAS considering all nucleotide substrings of length *k* (*k-mers*) to investigate host adaptation, niche gene associations and outbreak potential associated with distinct *C. jejuni* lineages.

## Results

### *C. jejuni* core and accessory genome analysis

Here we report on 490 genomes of *C. jejuni* isolated from samples of animal, human and environmental origins from two distinct continents. The average size of the *C. jejuni* genomes was 1 690 635 bp. We identified 1 111 core genes that covered 60% of the average *C. jejuni* genome size, while a set of additional 7 250 genes was identified in at least one of the genomes under consideration and therefore assigned to the accessory gene content.

### Core and accessory genome: phylogenetic structure and organisation of the *C. jejuni* population

**Figure 1:**
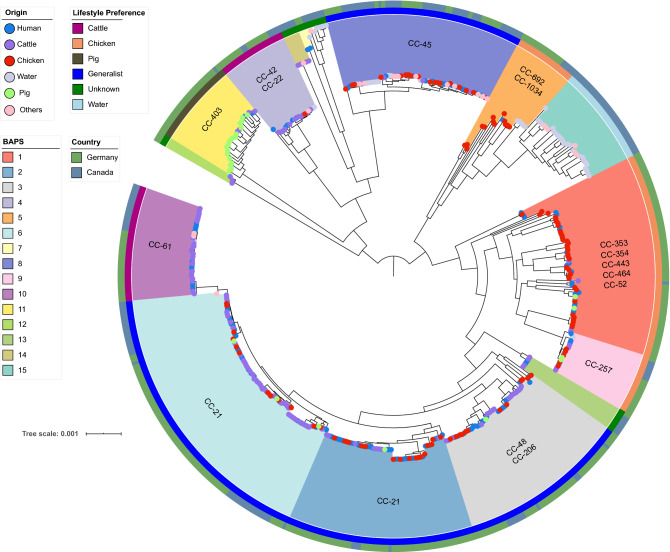
Population structure of *C. jejuni* based on the core genome alignment with BAPS clusters and clonal complexes colour-coded in the inner ring; Lifestyle preferences of the genomes coded in the second -ring; and country of genome origin described in the outer ring. The leaves are coloured by the origin of each sample.

The phylogenetic representation of the 490 core genomes showed 15 distinct phylogenetic branches (1–15) that have been confirmed by BAPS clustering (Fig. 1). BAPS clusters identified here, which comprised of more than 15 *C. jejuni* genomes, were further evaluated according to their respective CCs, original sample source and lifestyle classification (Table S1).

**Figure 2:**
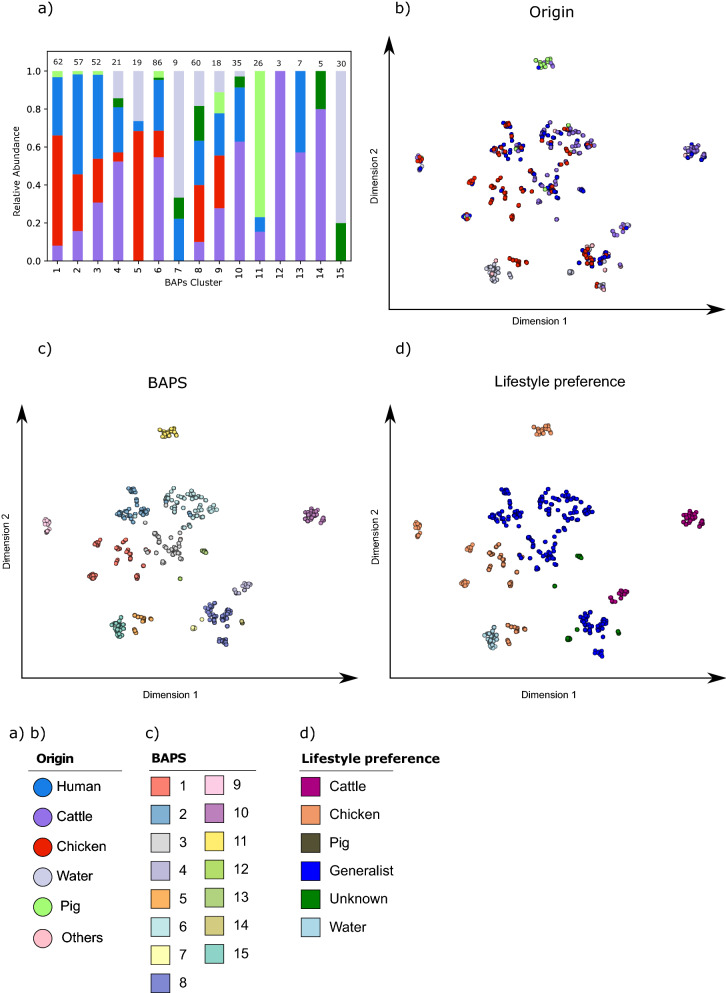
Relative distribution of sample origin among BAPS clusters and t-SNE plots of the accessory genome profile. **(a)** Shows the relative proportion of sample origins within the BAPS cluster that are later used for the stratified random sampling approach. **(b–d)** Show t-SNE plots in the 2-dimensional space of the accessory genome profiles. The colours included in the legend represent the sampling source, the BAPS clusters and the lifestyle preference are included in the legend.

For the original sample sources of the *C. jejuni* genomes investigated here, the relative proportion and absolute distribution for each of the BAPS clusters are visualised in Fig. 2a and supplementary Figure S1a. We identified a close phylogenetic relationship between genomes of BAPS cluster 5 representing the origin chicken with those of BAPS cluster 15 representing waterborne environmental *C. jejuni* (Fig. 1).

The genomes of BAPS cluster 15 and those of BAPS clusters with genomes from less than 15 isolates were not analysed with respect to their lifestyle preference and were therefore used as a control group in our study.

The lifestyle preference of each major BAPS cluster was determined and subjected to an internal assessment: As shown in Table S2, our assignments are generally concordant with lifestyle preferences reported for frequently occurring lineages such as CC-353, CC-354, CC-443, CC-464 and CC-52 (chicken), CC-42 and CC-61 (cattle) and CC-403 (pig). We also identified the probable lifestyle classification for the CC-22 lineage (cattle) and for isolates belonging to ST-2274 (chicken) (Table S1 and Table S2). Of note, the *C. jejuni* genomes associated with CC-21, CC-45 and CC-48 fulfilled the criteria for host-generalist lineages (Table S2).

Overall, the genomes assigned to individual BAPS clusters consisting of lineages considered as either host-specific for cattle (BAPS 4; including CC-42 and CC-22; BAPS 10, CC-61) or pigs (BAPS 11, CC-403) showed generally a less diverse population structure than those assigned to clusters associated with the host chicken (e.g. BAPS 5, including CC-1034 and CC-692). The distinct BAPS clusters comprising of host-generalist lineages (BAPS 8, including CC-45 and CC-283; BAPS 2, CC-21; BAPS 6, CC-21) showed a more diverse population structure (Fig. 1).

Our core genome-based phylogenetic analysis further revealed that cattle-related BAPS cluster 4 lineages (including CC-42 and CC-22) were more closely related to host-generalist lineages of BAPS cluster 6 with CC-21 than to other cattle-related lineages, for instance those of BAPS cluster 10 (Fig. 1). This also holds true for the chicken-related phylogenetic background (Fig. 1): While chicken-related BAPS cluster 1 was found being more closely related to BAPS cluster 6 of host-generalist lineage, BAPS cluster 5 showed less phylogenetic distance to BAPS cluster 8 (host-generalist lineage). These findings clearly reject the hypothesis of a common evolutionary background for host-specific lineages with respect to the host species represented here.

Minimum spanning trees based on MLST utilising BAPS cluster classification and lifestyle preferences are shown in the supplementary material (Figure S1). Finally, the accessory genome profiles of all genomes were visualised by t-SNE plots in Fig. 2b–d including sample origin, BAPS cluster and lifestyle preference. As expected, the overall population structure derived from the core genome is mirrored in the accessory genome content. Each BAPS cluster carries its unique set of accessory genes (Fig. 2c) confirming the population structure based on BAPS.

Also, *C. jejuni* genomes belonging to different BAPS clusters while sharing a particular lifestyle preference differ with respect to their accessory gene content (Fig. 2d). This observation is supported, for instance, by the accessory gene content identified for the cattle-specific BAPS clusters 4 and 10 (CC-42 and CC-61) and the chicken-specific BAPS clusters 1, 5 and 9 (CC-354, CC-692, CC-257, etc.) (Figs. 2c,d). Overall, BAPS clusters with a host-generalist lifestyle preference appear to have a broader gene pool within the accessory genome content than strains identified as host-specific.

### Recombination events in *Campylobacter jejuni* lineages

**Figure 3:**
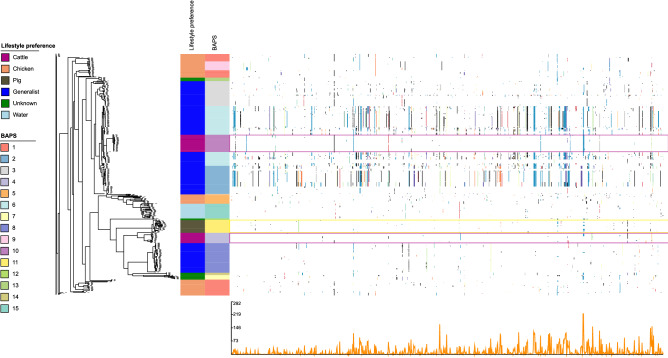
Recombination profile of the core genome alignment of 490 *C. jejuni* isolates calculated by BRATNextGen and visualized in Phandango. The left side shows the core genome phylogeny. The metadata provide information about lifestyle preferences (association) and BAPS clusters. Significant recombinations are marked by coloured dots and lines. Purple and yellow boxes highlight cattle- and pig-associated BAPS clusters, respectively. Presence of dot of the same colour across multiple isolates within a column represents acquisition of the same recombinant segment, otherwise colours are arbitrary. The line graph at the bottom presents recombination prevalence along the genome sequence.

Recombination events that show more differences between taxa than expected by mutation-driven evolutionary processes alone were illustrated in Fig. 3. Overall, CCs assigned as cattle- or pig-associated as well as those belonging to the group of host-generalists showed recombination profiles most likely resulting from intra-lineage genomic events. The pig-associated lineages of BAPS cluster 11 and the cattle-associated lineages of BAPS cluster 4 shared limited recombination patterns with other lineages and yielded a low recombination rate compared with other clusters, indicating the possible presence of lineage-specific recombination barriers (Fig. 3). The cattle-associated genomes forming BAPS cluster 10 showed several recombination events which were also indicated in the host-generalist lineages assigned to BAPS clusters 2, 3 and 6 (Fig. 3). However, the cattle-associated BAPS clusters 4 and 10 shared a single recombination site only. The host-generalist BAPS clusters 2, 3, and 6 were found being associated with more recombination events and some of these were shared by host-specific lineages, i.e. BAPS cluster 10 (cattle) and BAPS clusters 1, 5 and 9 (chicken), indicating genomic exchanges between these lineages. In addition, the analysis revealed that chicken-associated lineages (BAPS clusters 1, 5 and 9) were prone to trade off genetic material with each other and with host-generalist lineages (Fig. 3).

### In-silico identification of host-specific factors

After identifying significant *k-mers* using a consensus GWAS approach, the *k-mers* were mapped to an annotated reference genome in order to identify coding sequences (CDS) of the genome known to promote a particular lifestyle preference of *C. jejuni*^25^. A visualization of the resulting genes with corresponding p-values and frequencies for the matching *k-mers* are provided in supplementary Figure S2.

CDS identified by *k-mers* in the genomes of *C. jejuni* isolates with lifestyle preferences in pig and cattle showed a denser distribution around the expected allele frequency than the results obtained for the genomes representing chicken- or host-generalist lineages (Figure S2).

The genes identified by our analysis included accessory genes present in a limited number of genomic backgrounds and allelic variants of the core genome content. We identified several variants of core genes supporting specific lifestyle preferences in *C. jejuni*. To further evaluate the putative host-specific importance of the allelic variants identified, genes under consideration have been checked for non-synonymous base changes by comparing their predicted amino acid sequences. Several of these predicted aa sequences can be linked to particular lifestyle preferences of *C. jejuni* isolates. Details for all loci and aa sequence variants identified are provided in the Tables S3 (cattle), S4 (chicken), S5 (pig) and S6 (host-generalists).

### Accessory genes and allelic variants of the core genome associated with *C. jejuni* lineages assigned as pig-specific

In the genomes belonging to BAPS cluster 11 (CC-403) we identified 21,681 k*-mers* which are significantly associated with the host pig. These *k-mers* mapped to 49 accessory genes and 78 allelic variants of the core genome (Table S5). Considering the accessory genes, 14 were exclusively found within *C. jejuni* genomes from pig hosts. (Table 1). Three accessory genes (A6J90_06670, A6J90_06675, A6J90_02350) belonged to transcription units encoding type II restriction modification systems (RM systems), while a further gene encodes the restriction subunit (R) of the host specificity determinant (*hsd*R; A6J90_08990) of a type I RM system. Additional 8/14 genes were annotated as hypothetical or putative proteins without any specific functional information available in NCBI GenBank (17.06.2020).

**Table 1:** Selected accessory genes and allelic variants of the *C. jejuni* core genome content pig-associated.

Locus tag^a^	Gene	Predicted function	BfR- CA-14430^b^	COG^c^	COG^c^ description	Lifestyle preference^d^					Accessory/variant^e^
						Pig	Cattle	Chicken	Host generalists	Other	
						n	n	n	n	n	
A6J90_00190	–	Putative protein	–	–	–	25	0	0	0	0	A
A6J90_00195	–	Hypothetical protein	–	S	Function unknown	26	0	0	0	0	A
A6J90_00200	–	Hypothetical protein	–	–	–	26	1	0	0	0	A
A6J90_00270	–	Putative protein	–	–	–	26	0	0	0	0	A
A6J90_00275	*dpn*A	DNA methylase	–	L	Replication,recombination and repair	26	0	0	0	0	A
A6J90_01490	–	Putative protein	–	–	–	26	0	0	0	0	A
A6J90_01500/A6J90_01505	–	Hypothetical protein	–	V	Defense mechanisms	25	0	0	0	0	A
A6J90_02340	–	Undecaprenyl-diphos-phooligosaccharide-protein glycotransferase	–	–	–	25	0	0	0	0	A
A6J90_02350	–	R Pab1 restriction endonuclease	–	L	Replication, recombination and repair	25	0	0	0	0	A
A6J90_06670	–	Type II restriction endonuclease	–	L	Replication, recombination and repair	26	0	0	0	1	A
A6J90_06675	*hhaI*M	Cytosine-specificmethyl-transferase NlaX	–	H	Coenzyme transport and metabolism	26	0	0	0	1	A
A6J90_08990	*hsd*R	Type I restriction enzyme EcoR124II R protein	–	V	Defense mechanisms	26	0	1	0	0	A
A6J90_01640	–	Hypothetical protein	–	–	–	26	0	0	0	0	A
A6J90_02350	(*sua*5)	Hypothetical protein	–	J	Translation, ribosomal structure and biogenesis	26	0	0	0	0	A
Cj0321	*dxs*	l-Deoxy-d-xylulose-5-phosphate synthase	298.748	H	Coenzyme transport and metabolism	26	56	90	255	63	V
Cj1043c	*ten*I	Thiamine-phosphate Pyrophosphorylase	991.366	H	Coenzyme transport and metabolism	26	56	90	255	63	V
Cj1484c	-	Putative membraneprotein	1.428.185	–	–	26	56	90	255	63	V

^a^Locus tag for accessory genes based on *C. jejuni* reference genome CP022076.1 (NCBI accession). Locus tags for allelic variants of the core genome refer to *C. jejuni* strain NCTC11168 (NCBI accession: AL111168.1).

^b^Position of core genes in the reference strain BfR-CA-14430.

^c^Clusters of orthologous groups (http://clovr.org/docs/clusters-of-orthologous-groups-cogs/).

^d^Number of genomes assigned to a particular lifestyle carrying the gene or allelic variant (pig, cattle, chicken, host generalists, others).

^e^A indicates that a gene belongs to the accessory genome content of *C. jejuni*, while V indicates a specific allelic variant of the core genome content.

**Figure 4:**
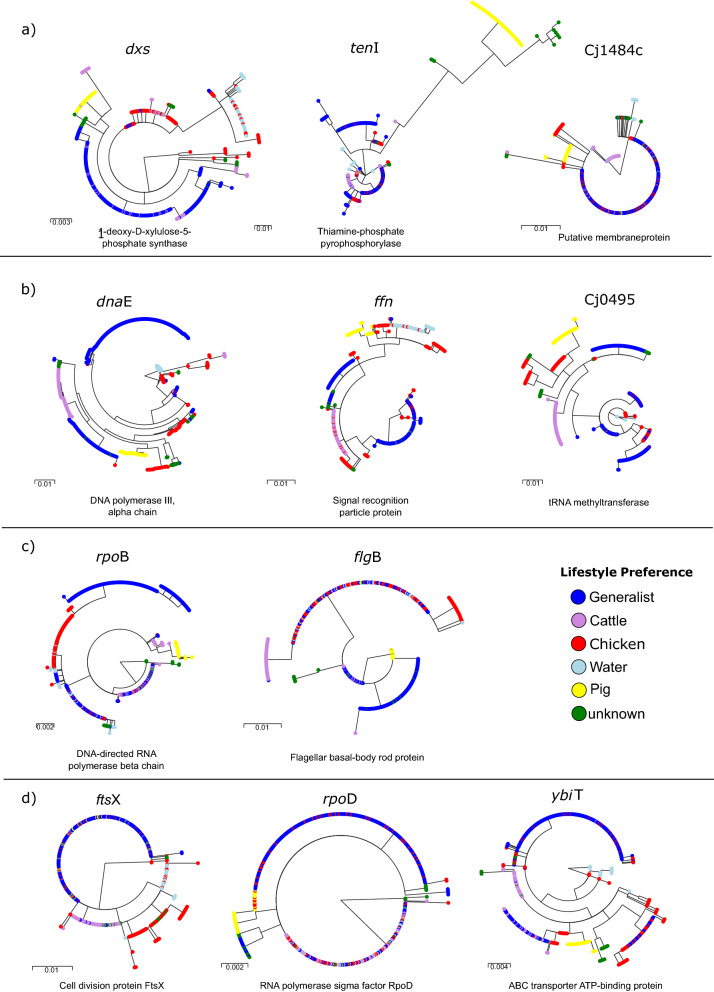
Phylogenetic tree of predicted amino acid sequence variants encoded by *dna*E, *ffh*, Cj0495, *rpo*B, *flg*B, *fts*X, *rpo*D, *ybi*T, *dxs*, *ten*I and Cj484c (selected from Tables 2, 3 and 4) that show lifestyle associated variants (colour coded in legend) in different phylogenetic lineages originating from different genetic and geographic backgrounds (Fig. 1).

Considering the *k-mer* results for genes belonging to the core genome, nucleotide changes leading to actual effects with respect to host adaptation capabilities of certain lineages are difficult to pinpoint. Here, we noted alterations for the predicted aa sequences associated with the capability of *C. jejuni* to synthesize vitamins and enzyme co-factors such as TenI and Dxs (Fig. 4a). In addition, the predicted aa sequence for Cj1484 was found to be altered (Fig. 4a).

### Accessory genes and allelic variants of the core genome associated with *C. jejuni* lineages assigned as cattle-specific

We further identified 66,491 k*-mers* for the cattle-associated genomes matching to 71 accessory genes and to 136 core gene variants (Table S3). According to our GWAS analysis, a particular accessory gene content which is representative for the lineages in both cattle-associated BAPS clusters (4 and 10) was not identified. However, 16 accessory genes were identified by *k-mers* significantly associated with CC-61 (BAPS cluster 10; Table S3). These genes belonged to a region of 9.9 kb size in *C. jejuni* (NCTC13261_01705 up to NCTC13261_01720). That particular locus contains 16 open reading frames encoding a HicA-HicB toxin/antitoxin system inhibiting the transfer of mRNA in case of nutrient limitation, a protein known to be involved in extracytoplasmatic stress response (YafQ) and regulatory protein RepA for plasmid DNA repair (Table S3).

Within the core genome we identified a 9.7 kb locus of 9 adjacent genes (Table 2) that encode for a ribosomal complex. While the allelic variants (non-synonymous substitutions) *dna*E and *ffh* (Fig. 4b) were identified as cattle-specific, identical variants of *ars*C, *aroF*, *ura*H, *rpl*S, *trm*D, *rim*M and *rps*P were identified in host-generalist BAPS cluster 8, too. However, for the genes *ura*H, *arsC*, *rpl*S and *rps*P, detected SNPs lead to synonymous changes only, indicating their biological importance as conserved housekeeping genes within the *C. jejuni* lineages investigated here.

**Table 2:** Selected accessory genes and allelic variants of the core genome content associated with the host cattle.

Locus tag^a^	Gene	Predicted function	BfR-CA-14430^b^	COG^c^	COG^c^ description	Lifestyle preference^d^					Accessory/variant^e^
						Pig	Cattle	Chicken	Host generalists	Other	
						n	n	n	n	n	
Cj0718	*Dna*E	DNA polymerase III, alpha chain	679,065	L	Replication, recombination and repair	26	56	90	255	63	V
Cj0717	*Ars*C	Putative ArsC family protein	678,288	P	Inorganic ion transport and metabolism	26	56	90	255	63	V
Cj0716	*Aro*F	Putative phospho-2-dehydro-3-deoxyhep-tonate aldolase	678,951	E	Amino acid transport and metabolism	26	56	90	255	63	V
Cj0715	*ura*H	Transthyretin-like periplasmic protein	676,514	S	Function unknown	26	56	90	255	63	V
Cj0714	*rpl*S	50S ribosomal protein L19	676,024	J	Translation, ribosomal structure and biogenesis	26	56	90	255	63	V
Cj0713	*trm*D	tRNA (guanine-N1)-methyltransferase	675,309	J	Translation, ribosomal structure and biogenesis	26	56	90	255	63	V
Cj0712	*rim*M	Putative 16S rRNA processing protein	674,773	J	Translation, ribosomal structure and biogenesis	26	56	90	255	63	V
Cj0710	*rps*P	30S ribosomal protein S16	674,308	J	Translation, ribosomal structure and biogenesis	26	56	90	255	63	V
Cj0709	*ffh*	Signal recognition particle protein	672,906	U	Intracellular trafficking, secretion, and vesicular transport	26	56	90	255	63	V
Cj0495	–	tRNA methyltransferase	465,764	J	Translation, ribosomal structure and biogenesis	26	56	90	255	63	V
Cj0017c	*dsb*I	Disulfid-deoxidoreductase	825,673	C	Energy production and conversion	26	56	90	255	63	V
Cj1233	–	HAD-superfamily hydrolase	1,175,101	S	Function unknown	26	56	90	255	63	V
_01705		Putative periplasmic protein	–			35	38	0	193	43	A
_01706	–	RelE/ParE family plasmid Stabilization system	–	S	Function unknown	35	20	0	0	4	A
_01707	*–*	Hypothetical protein	–		–	35	0	0	0	0	A
_01708	–	Hypothetical protein	–		–	35	0	0	0	0	A
_01709	–	Acyl carrier protein	–	K	Transcription	34	0	0	0	0	A
_01710	–	Hypothetical protein	–		–	35	0	0	0	0	A
_01711	*dna*G	DnaB-like protein helicase-like protein	–	L	Replication, recombination and repair	30	19	0	0	4	A
_01712	–	Hypothetical protein	–		–	34	7	0	1	4	A
_01713	–	Hypothetical protein	–		–	35	0	0	1	0	A
_01714	–	Helix-turn-helix domain-containing	–		–	35	19	0	1	4	A
_01716	–	Putative protein	–		–	35	0	0	0	0	A
_01717	*hic*B	Antitoxin HicB	–	S	Function unknown	34	14	0	1	4	A
_01718		Hypothetical protein	–	N	Cell motility	35	20	0	0	4	A
_01719	*hic*A	Probable mRNA interferase toxin HicA	–		–	35	20	0	0	4	A
_01720	–	Integrase	–	L	Replication, recombination and repair	35	20	0	1	4	A

^a^Locus tags for accessory genes based on *C. jejuni* reference strain NCTC13261 genome LR134500.1 (NCBI accession) while locus tags for allelic variants of the core genome refer to *C. jejuni* strain NCTC11168 (NCBI accession: AL111168.1).

^b^Position of core genes in the reference strain BfR-CA-14430.

^c^Clusters of orthologous groups (http://clovr.org/docs/clusters-of-orthologous-groups-cogs/).

^d^Number of genomes assigned to a particular lifestyle carrying the gene or allelic variant (pig, cattle, chicken, host generalists, others); ^e^ A indicates that a gene belongs to the accessory genome content of *C. jejuni* and V indicates a specific allelic variant of the core genome content.

Additional non-synonymous, cattle-specific allelic variants were also identified on independent positions within the genome, including the alleles Cj0495(Fig. 4b), *dsb*I and Cj1233 (Table 2).

### Accessory genes and allelic variants of the core genome associated with *C. jejuni* lineages assigned as chicken-specific

In comparison to the lineages associated with cattle, pig or even the host-generalists, chicken-associated lineages showed the broadest phylogenetic diversification in our study, mirrored by multiple lineages and CCs (Fig. 1), including enhanced divergence within a specific CC (CC-353 or CC-1034). Accordingly, this particular heterogeneity resulted in less host-specific signatures. The 5 712 chicken-associated *k-mers* identified by our GWAS analysis cover 17 accessory genes and 25 core gene variants (Table S4). A gene for a TraG-like protein of the type IV secretion system^26^ was detected among the accessory genomes in 59/90 chicken-associated genomes (Table 3). TraG-like proteins are known to play a crucial role in the conjugative transfer of plasmids^27^. Additionally, two genes for putative proteins of unknown function are carried by 66 and 68 of the chicken associated strains, respectively (Table 3).

**Table 3:** Selected accessory genes and allelic variants of the core genome content associated with the host chicken.

Locus tag^a^	Gene	Predicted function	BfR-CA-14430^b^	COG^c^	COG^c^ description	Lifestyle preference^d^					Accessory/variant^e^
						Pig	Cattle	Chicken	Host generalists	Other	
						n	n	n	n	n	
Cj0933c	*pyc*B	Putative pyruvate carboxylase B subunit	882.094	C	Energy production and conversion	26	56	90	255	63	V
Cj0478	*rpo*B	DNA-directed RNA polymerase beta chain	444.215	K	Transcription	26	56	90	255	63	V
Cj0528c	*flg*B	Flagellar basal-body rod protein	495.238	N	Cell motility	26	56	90	255	63	V
_01618	*Tra*G	Conjugal transfer protein TraG	–	U	Intracellular trafficking, secretion, and vesicular transport	1	1	59	1	13	A
_01627	–	Putative protein	–	–	–	3	0	66	1	7	A
_01633	–	Putative protein	–	–	–	3	0	68	0	0	A

^a^Locus tags for accessory genes based on *C. jejuni* reference strain NCTC13265 genome LR134498.1 (NCBI accession), while locus tags for allelic variants of the core genome refer to *C. jejuni* strain NCTC11168 (NCBI accession: AL111168.1).

^b^Position of core genes in the reference strain BfR-CA-14430.

^c^Clusters of orthologous groups (http://clovr.org/docs/clusters-of-orthologous-groups-cogs/).

^d^Number of genomes assigned to a particular lifestyle carrying the gene or allelic variant (pig, cattle, chicken, host generalists, others).

^e^A indicates that a gene belongs to the accessory genome content of *C. jejuni* and V indicates a specific allelic variant of the core genome content.

Like the cattle-associated lineages, chicken-associated genomes carry host-adapted allelic variants (Table 3). The allele encoding a specific aa variant of *rpoB* was identified in most of the genomes in all three chicken-associated BAPS clusters (Table 3, Fig. 4c). The gene variant encoding FlgB (Fig. 4c) is identical in BAPS clusters 1 and 5 (chicken) and the host-generalist BAPS cluster 2 (CC-21). Furthermore, a very closely related aa variant was identified in BAPS cluster 9 (chicken) as well. Additionally, the same allelic variant of the *pycB* gene is carried by most genomes of BAPS clusters 1 and 9 (Table 3).

### Independent adaptation of host-generalist lineages

Considering the core genome phylogeny of the *C. jejuni* strains presented here, the host-generalist lineages of BAPS cluster 8 appear to have evolved from independent genomic backgrounds, while other host-generalist lineages, for instance those of BAPS clusters 2, 3 and 6, appeared to be linked to each other (Fig. 1). In total, we have identified 37 339 k*-mers* which were mapped to 33 accessory genes and 87 core gene variants (Table S6). Accessory gene content exclusively associated with all host-generalist lineages was not identified by use of GWAS. A multitude of different allelic variants assigned to the core genome were identified for BAPS cluster 8 when compared with the genomes of the more closely related lineages of clusters 2, 3 and 6 (Table 4). Notably we also identified closely related variants for different core genes shared by all host-generalist lineages. These included *ftsX*, a gene involved in cell division, *ars*C, an arsenate reductase, further ribosomal genes (*rplS* and *rpsP*) and Cj0459c, known as a nicking endonuclease and purine-specific ribonuclease^28^ (Table 4). While the amino acid sequence encoded by *ftsX* shows a particular host-generalist-associated variant (Fig. 4d), the amino acid sequence determined by *ars*C, *rpl*S, *rps*P and Cj0459c are conserved in the *C. jejuni* population. Hence, *k-mers* identified for these CDS were associated with synonymous changes only. BAPS clusters 2, 3 and 6 harbour identical allelic variants for *dna*E and *ffh* (Fig. 4b). The same is true for several other genes such as *dxs*, *cys*M and *pck*A (Table 4) that are broadly distributed across the *C. jejuni* genome and are involved in multiple metabolic pathways. Additionally, genes involved in transcriptional pathways such as *rpoD* and substrate transport functions like *ybi*T (Fig. 4d) were identified.

**Table 4:** Selected allelic variants of the core genome content associated with host-generalists.

Locus tag^a^	Gene	Predicted function	BfR-CA-14430^b^	COG^c^	COG^c^ description	Lifestyle preference^d^					Accessory/variant^e^
						Pig	Cattle	Chicken	Host generalists	Other	
						n	n	n	n	n	
Cj1276c	*fts*X	Cell division protein FtsX	1.223.530	D	Cell cycle control, cell division, chromosome partitioning	26	56	90	255	63	V
Cj0459c	-	Conserved hypothetical protein (32.5% identical to HP0268)	428.984	–	–	26	56	90	255	63	V
Cj0321	*dxs*	1-deoxy-d-xylulose-5-phosphate synthase	296.904	H	Coenzyme transport and metabolism	26	56	90	255	63	V
Cj0912c	*cys*M	Belongs to the cysteine synthase cystathionine beta-synthase family	862.739	E	Amino acid transport and metabolism	26	56	90	255	63	V
Cj1001	*rpo*D	RNA polymerase sigma factor RpoD	945.528	K	Transcription	26	56	90	255	63	V
Cj0426	*ybi*T	ABC transporter ATP-binding protein	393.511	S	Function unknown	26	56	90	255	63	V
Cj0932c	*pck*A	Phosphoenolpyruvate carboxykinase (ATP)	880.507	H	Coenzyme transport and metabolism	26	56	90	255	63	V

^a^Locus tags for allelic variants of the core genome refer to *C. jejuni* strain NCTC11168 (NCBI accession: AL111168.1).

^b^Position of core genes in the reference strain BfR-CA-14430.

^c^Clusters of orthologous groups (http://clovr.org/docs/clusters-of-orthologous-groups-cogs/).

^d^Number of genomes assigned to a particular lifestyle carrying the gene or allelic variant (pig, cattle, chicken, host generalists, others).

^e^A indicates that a gene belongs to the accessory genome content of *C. jejuni*. Variant (V) indicates a specific allelic variant of the core genome content.

## Discussion

We show how the recently emerging research field of bacterial GWAS was able to identify genetic signatures that possibly play important roles for the host-specificity of *Campylobacter*. For each of the lifestyle preferences of *C. jejuni* investigated, we identified a broad set of allelic variants being associated with particular host-specific lineages from distantly related BAPS clusters, providing evidence for host-adaptive genetic signatures^29^.

We also extended the scheme of lifestyle preferences based on MLST to a whole genome level by applying BAPS and identified 15 distinct phylogenetic clusters. The efficiency of the proposed approach to identify lifestyle preferences by assigning host-specific or host-generalist *C. jejuni* lineages was verified by performing a comparison of the predicted lifestyles. For instance, CC-42 or CC-61 (cattle), CC-354 or CC-692 (chicken) and CC-403 (mammalian/pig) lifestyle assignments were verified with previously published reports on these *C. jejuni* lineages^8,30,31^. Additionally, putative novel lifestyle preferences of distinct lineages, i.e. CC-22 (cattle-specific) and ST-2274 (chicken-specific), were identified using the definition described above.

*C. jejuni* isolates assigned to either chicken or host-generalist lineages showed a diverse population structure, as reported before^32^. Contrarily, we found *C. jejuni* genomes identified as cattle-specific (CC-42 and CC-61) or pig-specific (CC-403) were less diverse and more clonal. Previous studies assumed that the tight clonal structure of the cattle-associated lineages CC-42 and CC-61 resulted from a more recent onset of the colonization of cattle by *C. jejuni* and therefore may reflect a bottleneck event in its evolution^24,29^. A similar host-adaptation process is possibly indicated by the limited diversity of CC-403 (pig-specific) assigned to BAPS cluster 11 in our study.

Genetic variation is known to be a pre-requisite to evolutionary change^33^. Since 2016, bacterial GWAS has advanced as a suitable method to identify genetic alterations associated with a phenotypical traits in large WGS datasets^34,35^, including studies on *C. jejuni*^13–16^. Acting like a “sieve”, genetic selection allows only a subset of mutations to persist and become an observable difference between genomes^33^. Allelic variants of *C. jejuni* core genes, independently acquired by different phylogenetic lineages leading to changes of known or predicted amino acid sequences, likely reflect adaptation to a particular ecological niche and/or host^36,37^. We have identified allelic variants of core genes which were clearly associated with the host species pig, cattle and chicken, even among distantly related BAPS clusters [BAPS 4 and 10 (cattle); BAPS 1, 5 and 9 (chicken)]. Further allelic variants (e.g. *ftsX* in CC-45 and CC-21) were identified as putative markers for host-generalist lineages. This observation is supported by the lack of notable recombination between CC-45 and CC-21^30^, indicating that these variants occurred independently of phylogenetic background and geographic origin. Therefore, mutant selection leading to homoplasy would be the most reasonable assumption. More research on the subject, including isolates covering a broader time span is needed to gain further insight into the bacterial evolution of *C. jejuni*.

For each of the CC-42, CC-22 and CC-61 cattle-associated lineages in BAPS cluster 4 and 10, a different set of specific accessory genes was identified. This may reflect independent colonisation events of that particular host in the evolutionary history of *Campylobacter*^38^. In BAPS cluster 10 we have identified genes associated with a HicA-HicB toxin/antitoxin system, which is suspected to inhibit the bacterial mRNA transfer in case of limited nutrient availability^39–41^.

Sharing the same host does not necessarily mean ample opportunities for DNA transfer with the host, since the preferred (sub-)niche of these CCs within the gut of cattle may differ, as it has been assumed for host-generalist lineages previously^30^. Furthermore, structure and composition of the gut microbiome may play a role, however little is known about the microbiome ecology and the putative lineage-specific differences among *C. jejuni* with respect to virulence-associated strategies such as attachment to host cell tissue^42,43^.

We identified a putative cattle-specific allelic variant of DNA polymerase III subunit alpha encoded by *dna*E, in which mutations have been shown to increase the overall mutation rate of *E. coli*^44,45^. Since an increased mutation rate is well known as a factor influencing niche adaptation^29^, the *dna*E variant may promote the host-specialization processes. In addition, we found cattle-specific changes of the gene encoding Ffh, a signal recognition particle protein (SRP). Ffh initiates the co-translational targeting of membrane and secretory proteins to the cytoplasmic bacterial membrane^46^, indicating adaptation of transport processes. In *E. coli*, the SRP system plays an important role in membrane protein biosynthesis, and previous research also indicated that Ffh is involved in the regulation of membrane protein translation^47^. Notably, a GTPase (FlhF) possessing an active domain most similar to Ffh, was found to be involved in flagellar gene regulation and biosynthesis in *C. jejuni*^48^. Again, the lack of corresponding recombination patterns indicated that niche-specific environmental pressure induced the predicted amino acid change of Ffh independently in distantly related lineages as we demonstrated in Fig. 3. Indeed, *ffh* has already been described as a homoplasic gene on a nucleotide level in cattle-associated *C. jejuni* genomes by a recent study^24^.

Most of the CC-403 and ST-1942 (pig-associated) *C. jejuni* in BAPS cluster 11 carry a unique set of genes encoding restriction modification (RM) systems (RM I and RM II) that may contribute to lineage-specific barriers shielding the bacteria from intrusion of foreign DNA, a phenomenon reported before^49–51^. As well, the frequency and pattern of intra-lineage recombination events was unique to CC-403 and its related STs, as noted before^52^. However, due to the limited number of pig-associated clades, particular differentiation between a lineage or host specific association is challenging.

While amino acid variants encoded by the *tenl* gene is thought to affect the thiamine metabolism and may serve as markers for cattle-specific niche adaption^24^, in this study we identified pig-specific variations as well. The amino acid changes associated with the allelic variant encoding final aromatase (TenI) needed in thiamine biosynthesis were extensive and may indicate functional alterations or even loss-of-function. Further research to characterise this gene would be useful for potential agrifood intervention strategies. Since industrial diets for pigs are generally supplemented with thiamine^53^, reduction or even shutting-off the metabolic pathway might conserve energy and seems therefore beneficial for pig-specialized *C. jejuni* lineages. In addition, we identified a pig-specific variant of the putative thiamine-dependent synthase encoded by *dxs*, again underlining the general importance of specific alterations of the thiamine pathway for host adaptation of *C. jejuni* lineages. The majority of the accessory genome assigned in this study as chicken-specific included, among others, genes for a putative conjugative transfer protein (*Tra*G-like), which is commonly linked to a type IV secretion system essential for DNA transfer in bacterial conjugation^54,55^. These findings are in concordance with the recombination analysis for the chicken-specific lineages (e.g. CC-257 or CC-354), which indicated multiple horizontal gene transfer events. With respect to *k-mers* that indicate sequence alterations of the core genomes and lead to aa variants of the respective proteins, we noted significant *k-mers* mapping to the gene encoding PycB, the second subunit of the anaplerotic and glucogenic pyruvate carboxylase in *C. jejuni*^56^. This finding indicates a specific adaptation of a basal metabolic pathway in *C. jejuni*. In addition, we detected significant *k-mers* associated with a *rpo*B variant, a housekeeping gene used for investigating genetic relatedness within the *Campylobacter* genus^57^. Interestingly, several different mutations of *rpoB* enhance growth at 42.2 °C compared to the wildtype in *E. coli*^58^. Since the body temperature of poultry is commonly between 39 and 43 °C^59^, the *rpoB* variant might contribute to temperature–induced adaptive changes *in C. jejuni*.

The large host-generalist lineages belonging to either BAPS clusters 2, 3, 6 (CC-21/CC-48/CC-206) or BAPS cluster 8 (CC-45) showed clear differences concerning their accessory gene content, an observation confirmed by earlier results from Yahara et al., who tracked these lineages from the chicken flock through the meat production chain as well as in clinical samples of human origin^14^. Here, we have provided evidence that accessory gene patterns were mostly BAPS clusters-specific, irrespective of the sample origin (e.g. animal, human clinical or environment). Host-generalist BAPS clusters appear to possess a larger pool of accessory genes, possibly indicating a repertoire of genes promoting survival in different hosts and environments^60,61^. This idea is supported by our recombination analysis, showing that host-generalist lineages are prone to DNA exchange, thus, natural transformation and recombination between host-generalist lineages enhances adaptive possibilities needed to survive in different hosts.

Variation of predicted aa sequences possibly associated with a host–generalist lifestyle of specific *C. jejuni* lineages were, for instance, identified for the cell division protein encoded by *fts*X. Recent work by Riedel et al. showed that *fts*X transcription is downregulated in *Campylobacter lari* after exposure to heat stress^62^, possibly indicating certain allelic variants may differ with respect to their stress response. As mentioned earlier, allelic variants may have evolved individually in both lineages (CC-45 and CC-21/CC-48), since the recombination analysis suggests a limited number of recombination events between BAPS clusters 8 and 2, 3 and 6.

Distinct host-specific factors, such as body temperature, the structure and composition of the gut microbiota, mucosal structures and immune system shape the adaptation strategies of *C. jejuni* lineages. Focusing fundamental science research in these areas will enhance the opportunity to exploit this foodborne pathogen’s ability to thrive in niche environments with targeted intervention strategies in the future.

## Material and methods

### Strain selection and genome sequencing

A uniform stratified random collection comprising 324 *C. jejuni* isolates obtained from samples of four different species, including human (n = 96), chicken (n = 102), cattle (n = 98) and pig (n = 28). The original samples were collected in 16 different federal states in Germany, between 2010 and 2019. Isolates from healthy and diseased animals as well as human clinical isolates were included (Table S1). The animal-derived isolates were provided by the National Reference Laboratory for *Campylobacter* at the German Federal Institute for Risk Assessment (BFR) and the Institute of Microbiology and Epizootics (IMT) at Freie Universität Berlin, while the human-derived isolates were provided by the National Reference Centre for *Salmonella* and other Bacterial Enterics at the Robert Koch Institute (RKI). *C. jejuni* is rarely isolated from porcine, therefore porcine-derived isolates were limited. In order to limit spatial and temporal effects, the set of genomes investigated here was complemented by whole genome data of further 166 isolates from a Canadian study which included *C. jejuni* from cattle (n = 39), chicken (n = 12), human clinical cases (n = 40), environmental (n = 54) and other animal (n = 21) origins^16^. The original purpose of the Canadian study was to identify diagnostic markers which can be used for rapid screening approaches to detect *C. jejuni* subtypes^16^. The complete list of all 490 genomes, including available metadata such as sample origin/source and baseline typing data such as ST is provided in Table S1. Detailed protocols used for whole genome sequencing (WGS) are provided as supplementary material. Illumina raw read data sequenced for this study is available at the National Center for Biotechnology Information (NCBI) under Bioproject ID PRJNA648048. Furthermore we included the strain BfR-CA-14430, available at NCBI under the accessory numbers CP043763.1 and CP043764.1, already published as a representative *C. jejuni* genome by the zoonosis monitoring program of Germany^63^.

### Assembly and annotation

The Illumina paired-end reads were adapter-trimmed by Flexbar v.3.0.3^64^ and corrected using BayesHammer^65^. The de novo assembly was performed using SPAdes v3.11.1^66^ with default settings. All genomes were annotated by Prokka v1.13^67^ employing a customized database which consist of 137 complete annotated reference genomes provided by NCBI as described before^63^.

### Multilocus sequence type (MLST) analysis

In silico MLST was carried out on seven housekeeping genes (*aspA, glnA, gltA, glyA, pgm, tkt, uncA*) as described by Dingle et al.^32^. This was done with the BLAST-based tool “mlst” (https://github.com/tseemann/mlst) based on the *Campylobacter jejuni*/*coli* database of pubmlst^68^. Obtained MLST profiles were then used to calculate a minimum spanning tree by MSTree V2 that was visualized with GrapeTree^69^.

### Pan-genome and phylogenetic analyses

Open reading frames (ORFs) predicted by Prokka were subsequently used as input for Roary v3.12.0^70^ to calculate the pan-genome size and core genome alignment using default settings. The resulting alignment was used to calculate a maximum likelihood-based phylogeny with RAxML v.8.2.10^71^ with 100 bootstraps under the assumption of the gtr-gamma DNA substitution model^72^. ClonalFrameML v1.11^73^ was used to correct for recombination events and phylogenetic groups were identified with Bayesian Analysis of Population Structure (BAPS). Here, we used BAPS with hierarchical clustering that was implemented in the R packages RhierBAPS v1.0.1^74^. Grouping of the accessory genome was further analysed by t-distributed stochastic neighbour embedding (t-SNE)^75^.

### Recombination analysis

BratNextGen^76^ was used to reconstruct putative recombination events based on the analysis of the core genome alignment of our selection comprising 490 *C. jejuni* genomes. Parameter estimation was performed based on 20 iterations and significant recombinations (p-value $\le$ 0.05) were obtained using permutation testing with 100 permutations executed in parallel.

### Genome-wide association study (GWAS)

In order to perform an in-depth analysis of genomic alterations possibly associated with host specificity, pyseer v.1.1.2^25^ was used for GWAS based on variable-length *k-mer* composition (9 to 100 base pairs) for all 490 genomes. To control the lineage-level associations reported for bacterial GWAS (Earle et al., 2016; PMID: 27572646) a linear mixed model (LMM) has been integrated (details are provided by the supplementary section on GWAS ). *K-mers* significantly representing distinct isolate origins (human, cattle, chicken or pig) were further mapped by bwa v0.7.17^77^ against selected reference genomes from this study set in order to identify putative origin-specific factors, genes and consecutive gene loci.

In order to reduce the false positive rate of the GWAS and account for highly unbalanced groups, we employed a bootstrapping approach. Further details can be found in the supplementary material.

The consequential set of genes was further analysed considering functional annotations and metabolic pathways using EggNog v.4.5.1^78,79^.

### *C. jejuni* lifestyle classification

In order to facilitate statistical comparison, we adapted a definition from Shepard et al.^30^ and defined a set of closely-related *C. jejuni* lineages as host-specific if ≥ 50% genomes building the respective BAPS cluster were associated with isolates from a specific animal origin (e.g. cattle, chicken) while each of the other isolate origins contributed less than 10% in the BAPS cluster. Potential host-generalist lineages were assumed when more than 25% of the clustering genomes represented in the corresponding BAPS cluster were from *C. jejuni* of human clinical cases while at least two further animal origins account for more than 10% of the remaining genomes, respectively.

## Supplementary Information

Supplementary Tables.

Supplementary Information.
